# Decreasing Emergency Room Utilization in High Risk Geriatric Patients

**DOI:** 10.1093/geroni/igaa057.443

**Published:** 2020-12-16

**Authors:** Shamsi Fani, Lizette Munoz, Susana Lavayen, Blair McKenzie, Audrey Chun, Jeff Cao, Stephanie Chow

**Affiliations:** 1 Mount Sinai Medical Center, New York, New York, United States; 2 Mount Sinai Hospital, New York, New York, United States; 3 The Mount Sinai Hospital, New York, New York, United States; 4 Icahn School of Medicine at Mount Sinai, New york, New York, United States; 5 Icahn School of Medicine at Mount Sinai, New York, New York, United States

## Abstract

Background: The Acute Life Interventions Goals & Needs Program (ALIGN) at the Mount Sinai Hospital in New York City aims to work closely with high risk geriatric patients for short term intensive management of acute medical and social issues. Quantitative measures for determining success of the program is comparing emergency room visits and hospitalizations prior to and after enrollment with ALIGN. The Community Paramedicine service allows a paramedic, the ALIGN provider, and an emergency room physician to assess and triage patients in their home via video conference thereby avoiding ED visits for non-urgent services. Method: We reviewed the utilization of the Community Paramedicine service (from July 2017-February 2020) and its impact on ALIGN’s efforts to reduce unnecessary ED visits and hospitalizations. Results: 36 patients were evaluated with the Community Paramedicine service (from July 2017-February 2020). 19 or 52.8% avoided an ED visit and 17 or 47.2% were transported to the ED. 12 or 70.6% were admitted to the hospital of those that were transported to the ED initially. Top reasons for transport to ED included generalized weakness, acute mental status change (AMS), and shortness of breath (SOB). Conclusions: A Community Paramedicine program utilized by a high risk geriatrics team like ALIGN is effective in reducing ED visits and hospitalizations for the elderly population who incur greater expenses to the health care system and traditionally have poorer health outcomes.

